# Eccentric Strength Assessment of Hamstring Muscles with New Technologies: a Systematic Review of Current Methods and Clinical Implications

**DOI:** 10.1186/s40798-021-00298-7

**Published:** 2021-01-28

**Authors:** João Gustavo Claudino, Carlos Alberto Cardoso Filho, Natália Franco Netto Bittencourt, Luiz Gilherme Gonçalves, Crislaine Rangel Couto, Roberto Chiari Quintão, Guilherme Fialho Reis, Otaviano de Oliveira Júnior, Alberto Carlos Amadio, Daniel Boullosa, Júlio Cerca Serrão

**Affiliations:** 1 School of Physical Education and Sport – Laboratory of Biomechanics, Universidade de São Paulo, Av. Prof. Mello de Morais, 65 – Cidade Universitária, São Paulo, São Paulo 05508-030 Brazil; 2 Research and Development Department, LOAD CONTROL, Contagem, Minas Gerais Brazil; 3 Uni-BH University Center – Physical Therapy Department, Belo Horizonte, Minas Gerais Brazil; 4 PHAST, Belo Horizonte, Minas Gerais Brazil; 5 Physiology Department, Botafogo Futebol Clube, Ribeirão Preto, São Paulo Brazil; 6 Universidade Federal de Minas Gerais, Belo Horizonte, Minas Gerais Brazil; 7 Medical Department, Clube Atlético Mineiro, Belo Horizonte, Minas Gerais Brazil; 8 Sport and Exercise Science, College of Healthcare Sciences, James Cook University, Townsville, QLD Australia; 9 Graduate Program in Movement Sciences, INISA, Universidade Federal de Mato Grosso do Sul, Campo Grande, Mato Grosso do Sul Brazil

**Keywords:** Knee flexors, Muscle injury, NordBord, Flywheel, Screening, Neuromuscular status, Strain, Athletic performance

## Abstract

**Background:**

Given the severe economic and performance implications of hamstring injuries, there are different attempts to identify their risk factors for subsequently developing injury prevention strategies to reduce the risk of these injuries. One of the strategies reported in the scientific literature is the application of interventions with eccentric exercises. To verify the effectiveness of these interventions, different eccentric strength measurements have been used with low-cost devices as alternatives to the widespread used isokinetic dynamometers and the technically limited handheld dynamometers. Therefore, the purpose of the present systematic review was to summarize the findings of the scientific literature related to the evaluation of eccentric strength of hamstring muscles with these new technologies.

**Methods:**

Systematic searches through the PubMed, Scopus, and Web of Science databases, from inception up to April 2020, were conducted for peer reviewed articles written in English, reporting eccentric strength of hamstrings assessed by devices, different to isokinetic and handheld dynamometers, in athletes.

**Results:**

Seventeen studies were finally included in the review with 4 different devices used and 18 parameters identified. The pooled sample consisted of 2893 participants (97% male and 3% female: 22 ± 4 years). The parameters most used were peak force (highest and average), peak torque (average and highest), and between-limb imbalance (left-to-right limb ratio). There is inconsistency regarding the association between eccentric hamstrings strength and both injury risk and athletic performance. There is no standardized definition or standardization of the calculation of the used parameters.

**Conclusions:**

The current evidence is insufficient to recommend a practical guide for sports professionals to use these new technologies in their daily routine, due to the need for standardized definitions and calculations. Furthermore, more studies with female athletes are warranted. Despite these limitations, the eccentric strength of hamstring muscles assessed by different devices may be recommended for monitoring the neuromuscular status of athletes.

**Supplementary Information:**

The online version contains supplementary material available at 10.1186/s40798-021-00298-7.

## Key Points

- Eccentric hamstrings strength using 4 different devices was evaluated with 18 different parameters after 3-to-6 trials performed unilaterally or bilaterally. Peak force, peak torque, and between-limb imbalance were the most used parameters.
- Eccentric strength hamstring assessed by different devices can be a useful tool for monitoring neuromuscular status of athletes in laboratory and field settings. The assessments should be performed bilaterally with the average of 6 trials.
- There is a need of consensus for identifying the best procedures, definitions, and calculations for evaluation of the eccentric strength of hamstrings in different sport settings.

## Background

Despite the constant evolution of applied sport training tools and technologies, including 24-h monitoring [1] and artificial intelligence [2], hamstring strain injuries are still the most common injuries in sports involving high-speed running activities [3]. A longitudinal analysis between 2001 and 2013 found that hamstring injuries had annually increased by 4% in professional soccer [4]. In addition to soccer [5], hamstring injuries are common in other team sports as American Football [6], Australian Rules Football [7], basketball [8], cricket [9], rugby [10], and in individual sports as track and field [11].

Eccentric exercises are one of the most popular strategies with strong evidence supporting their use for injury prevention [3]. Given the severe economic and performance implications of hamstring strain injuries, there are attempts to identify the main risk factors associated with these injuries and to develop efficient prevention strategies [3]. In this context, new alternative tools to isokinetic and handheld dynamometers [12, 13], with requirements of minimal equipment and easy-to-use in the field, have been developed to verify the effect of eccentric exercises on different eccentric strength measures [14, 15]. Previously, Tous-Fajardo et al. [14] tested an instrumented flywheel leg-curl machine that offered eccentric overload for hamstring development. Opar et al. [15] developed a novel field-testing device, the NordBord, for assessing hamstring eccentric strength, based on the commonly employed Nordic hamstring exercise. These devices have received an increasing interest from practitioners and researchers [16] and many other interesting field applications to reduce hamstring injuries are currently being published [15–18].

However, there is no evidence to support the decision-making process of practitioners for using these new devices. Therefore, the purpose of the present systematic review was to search, analyze, and summarize the current findings of the scientific literature related to the evaluation of eccentric strength of hamstring muscles with these new technologies.

## Methods

We adopted the Preferred Reporting Items for Systematic Reviews and Meta-Analyses (PRISMA) guidelines [19].

### Sources and Study Selection Process

Three electronic databases (PubMed, Scopus and Web of Science) were systematically searched from inception up to April 2020. The command line (“eccentric strength” OR “Nordic exercise” OR “hamstring”) AND (“equipment” OR “device”) was used during the electronic searches. Titles and abstracts were reviewed and screened by the first author (JGC) for the potential eligible studies based on inclusion criteria. Two authors (CACF and NFNB) retrieved and independently assessed the full text of the potential eligible studies. If any doubt arose during this process, a fourth author (JCS) was involved for the final decision by consensus.

### Eligibility Criteria

1. The study was written in English.
2. The study was published as original research in a peer-reviewed journal as a full text article.
3. Data were reported from team or individual sport athletes.
4. The participants were competitive athletes (defined as Olympic, international, professional, semi-professional, national, regional, youth academy or division I collegiate);
5. Eccentric knee flexors strength was assessed by devices different to isokinetic and handheld dynamometers.

### Data Extraction

Three review authors (JGC, CACF, and NFNB) independently extracted information from the included full-text publications, such as authors, year, population information (mean age, sex ratio, sample size, sport, competitive level), study design, devices used to assess eccentric hamstrings strength, data collection procedures (number of repetitions, performed bilaterally or unilaterally), and the main findings of these studies about injury risk or sports performance. Discrepancies were resolved through discussion until consensus was reached. A narrative synthesis of data was performed.

### Quality Assessment

The quality of all studies was evaluated by two authors (LGG and CRC) using evaluation criteria (Table 1) based on a study by Saw et al. [20]. Scores were allocated based on how well each criterion was met, assuming a maximum possible score of 8 (low risk of bias). Studies with a risk of bias score of 4 or less were considered poor and were subsequently excluded. Once the studies to be included had been defined, we performed an additional review, checking reference lists [21] to identify additional peer reviewed studies that met the inclusion criteria. One study which met inclusion criteria was also included during the peer review process following a reviewer suggestion.

**Table 1:** Risk of bias assessment criteria

Criteria		Definition	Scoring		
			0	1	2
A	Peer reviewed	Study published in peer-reviewed journal	No	Yes	–
B	Number of participants	Number of participants included in study findings	< 5	6–30	> 31
C	Population defined	Age, sex, sport, time experience (or level) were described	No	Partly	Yes
D	Experimental design	Experimental design of the study period was described and replicable	No	Partly	Yes
E	Eccentric strength parameters	The eccentric strength parameters of hamstring assessed by devices were described	No	Yes	–

## Results

**Fig. 1:**
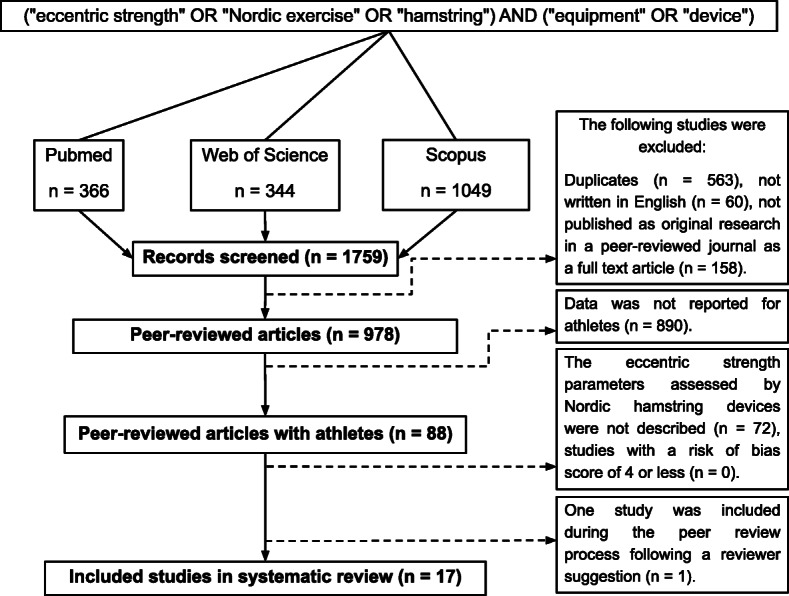
Study selection PRISMA flow diagram

The initial search returned 1759 articles (see Fig. 1). After the removal of duplicates (*n* = 563), a total of 1196 studies were retained for full text screening. Following the eligibility assessment, 1180 studies were excluded as they did not meet the inclusion criteria. Finally, after considering one study suggested by one of the reviewers, 17 studies were included in this systematic review [14–16, 22–35].

### Characteristics of the Studies and Risk of Bias

The pooled sample size and age included 2893 participants with 22 ± 4 years, being composed mostly by male athletes (97%). The athletes were involved in only seven sports: soccer (70%), Australian Rules Football (12%), rugby (7%), alpine skiing (6%), cricket (1%), and track and field (sprinters) (1%). Three studies did not report the sport of the athletes (3%). All the studies included had a low risk of bias, with a score > 4 (see online supplementary Table 1). The average bias score for the studies was 7.6 (range 5–8).

There were 16 observational studies, with 9 cross-sectional studies [14, 16, 22, 24–27, 34, 35], 6 prospective cohort studies [23, 28–32] and one reliability and case-control study [15]. The follow-up period of these prospective cohort studies ranged between one preseason period [30] to four seasons [23]. An interventional cross-over study [33] was also included.

### Main Findings

The summary of the 17 studies included in the systematic review is provided in Table 2. Four different devices were found to assess eccentric strength of hamstrings: an instrumented flywheel leg-curl machine (6%) [14], the NordBord (82%) [15, 16, 22–24, 26, 28–35], a custom-made device based on the prototype validated by Opar et al. [13] with two commercially-available load cells (6%) [25], and a custom-made device with load cells fixed in the dominant leg with a built-in potentiometer to measure sagittal knee angle (6%) [27].

**Table 2:** Summary of studies

Study	Sport (profile)	Experimental design	Main findings
2006 Tous-Fajardo et al. [14]	Soccer and Rugby (*n* = 20; M) Age: 26 ± 4 y Age: 25 ± 3 y	Cross-sectional study	**1)** The flywheel leg-curl device offered eccentric overload in a range of motion near complete extension about the knee joint. The magnitude of this overload is related to the athlete’s training background; in other words, the athletes which had previous experience using this novel technology showed greater eccentric strength performances than novice athletes of the same caliber. It was observed that biceps femoris muscle plays a more critical braking role than semitendinosus. **Device:** YoYo (Technology AB, Stockholm, Sweden) instrumented with strain gauge (MuscleLab Force Sensor) to measure the force. **Parameters:** peak force (N); average force (N); peak power (W); average power (W); peak velocity (m/s); average velocity (m/s). The data was recorded from 6 coupled concentric-eccentric actions. Parameters’ definition was not reported (bilateral analysis).
2013 Opar et al. [15]	Australian Football, Rugby, Soccer and Sprinting (*n* = 50; M) Age: not reported Elite and sub-elite	Reliability and case-control study	**2)** The experimental device to assess eccentric knee flexor strength showed high to moderate test-retest reliability for measurements when the Nordic hamstring exercise was performed bilaterally (ICC = 0.83–0.90 and SEM = 6–9%), but less reliability during unilateral testing (ICC = 0.56–0.73 and SEM = 10–11%). Moreover, between elite athletes who had a unilateral history of hamstring strain injury within the previous 12 months, there was a significant eccentric knee flexor weakness in their injured limb compared to their uninjured limb and to the limbs of uninjured recreational athletes. **Device:** a novel device using uniaxial load cells (MLP-1 K; Transducer Techniques, Inc., Temecula, CA; NordBord prototype). **Parameters:** peak force (average); the mean of the peak forces from 6 trials of Nordic hamstring exercise in absolute terms (N). Peak force (highest); the best of 6 trials of Nordic hamstring exercise in absolute terms (N), for each limb (left and right). For previously injured players, only the average peak force based on 6 bilateral trials was determined. Between-limb imbalance ratio was measured as the left-right limb ratio. These between-limb ratios were them converted to the percentage difference using log-transformed raw data followed by back transformation (bilateral and unilateral analyses).
2015 Bourne et al. [29]	Rugby (*n* = 194; M) Age: 23 ± 4 y Elite, sub-elite, and U19	Prospective cohort studies study	**3)** Lower eccentric knee flexor strength was not associated with an increased risk of hamstring strain injury (HSI). But, higher levels of between-limb imbalance (≥ 15%) were associated with a significantly increased risk of a subsequent HSI, and this was amplified in athletes who had suffered the same injury in the previous 12 months. **Device:** a custom-made uniaxial load cells (Delphi Force Measurement, Gold Coast, Australia; NordBord prototype). **Parameters:** peak force (highest); the best of 3 repetitions of Nordic hamstring exercise for each limb (left and right), in absolute terms (N) and relative to body weight (N/kg). The between-limb imbalance was calculated as left/right limb ratio, for the uninjured group and as uninjured/injured limb ratio, for the injured group. These between-limb ratios were them converted to the percentage difference using log-transformed raw data followed by back transformation. In some analysis, to have a single measure of eccentric hamstring strength for each athlete, an averaging the peak forces from each limb (2-limb-average strength) was made (unilateral and bilateral analysis).
2015 Opar et al. [28]	Australian Football (*n* = 210; M) age: 23 ± 4 Elite	Prospective cohort studies study	**4)** Lower limbs that sustained a HSI were weaker than uninjured limbs at the start and end of preseason. Eccentric hamstring strength below 256 N at the preseason start and below 279 N at the end of preseason increases the risk of HSI. The between-limb imbalance in eccentric hamstring strength was not different between the injured and uninjured groups. And finally, the interaction between athlete age, history of hamstring strain injury, and eccentric hamstring strength provided better information on athlete’s injury risk profile than only athlete’s age and history of HSI. **Device:** a custom-made uniaxial load cells (Delphi Force Measurement, Gold Coast, Australia; NordBord prototype). **Parameters:** peak force (average); the mean of the peak forces from 3 trails at Nordic hamstring exercise for each limb (left and right) resulting in a left and right limb measure, reported in absolute terms (N) and relative to body weight (N/kg). For the athletes that remained injury free, the strength measurement of right and left limb was averaged. The between-limb imbalance was calculated as left/right ratio for uninjured group and as an uninjured/injured limb ratio in the injured group; these between limb ratios were them converted to the percentage difference using log-transformed raw data followed by back transformation (unilateral and bilateral analysis).
2015 Opar et al. [30]	Australian Football (*n* = 99; M) Age: 23 ± 3 y Elite	Prospective cohort study	**5)** Athletes with a unilateral history of HSI's displayed smaller increases in eccentric hamstring strength compared with the control group athletes, who had no history of injury, during a preseason training. Interestingly, the smaller increase in eccentric strength across the preseason was not restricted to the previously injured limb, as the contralateral limb displayed small increases too, which might suggest that the effects of a prior HSI may be centrally mediated. **Device:** a custom-made uniaxial load cells (Delphi Force Measurement, Gold Coast, Australia; NordBord prototype). **Parameters:** peak force (average); the mean of the peak forces from 3 contractions during Nordic hamstring exercise for each limb (left and right) in absolute terms (N) and relative to early preseason strength measure expressed as the late preseason/early preseason ratio. In some analysis, to have a single measure of eccentric hamstring strength for each athlete, an averaging the peak forces from each limb (2-limb-average strength) was made (bilateral and unilateral analysis).
2016 Buchheit et al. [16]	Soccer and Australian Football (*n* = 122; M) Age: 22 ± 7 y Elite, 4th Division, U21, U19, and U17	Cross-sectional study	**6)** Eccentric knee flexor strength, as assessed with the NordBord device is largely body mass dependent, but simply dividing eccentric strength by units of BM (i.e., N/Kg) may not be an optimal strategy. To control body mass (BM) effect, practitioners may compare actual test performances with the expected strength for a given BM, using the following predictive equation: eccentric strength (N) = 4 × BM (kg) + 26.1. Value deviating from body mass expected values by last 40 N (12%) may be considered greater or lower. **Device:** a custom-made uniaxial load cells (Delphi Force Measurement, Gold Coast, Australia; NordBord prototype). **Parameters:** peak force (highest); the best of 3 repetitions of Nordic hamstring exercise for right and left limbs expressed in absolute terms (N). In the between-limb imbalance, the average strength of left and right legs was used for analysis (bilateral analysis).
2016 Timmins et al. [31]	Soccer (*n* = 152; M) Age: 25 ± 5y Elite	Prospective cohort study	**7)** Athletes with biceps femoris long head fascicle length (BFlh) shorter than 11 cm were 4.1 times more likely to suffer a HSI, and low levels of eccentric knee flexor strength (below 337 N) increases in 4.4 times the risk of HSI. For every increase in eccentric knee flexor strength, the injury risk was reduced by 9%. No measure of MVIC strength or between-limb imbalances (eccentric strength and BFlh fascicle) led to a statistically increase in HSI risk. **Device:** a custom-made uniaxial load cells (Delphi Force Measurement, Gold Coast, Australia; NordBord prototype). **Parameters:** peak force (average); the mean of the peak forces from 3 contractions during Nordic hamstring exercise for each limb (left and right), reported in absolute terms (N) and relative to body weight (N/kg). Peak torque (average); the product of peak force (average) by the shank length (m) for each limb (left and right), reported in absolute terms (Nm) and relative to body weight (Nm/kg). The shank length (m) was determined as the distance from the lateral tibial condyle to the mid-point of the brace that was placed around the ankle during Nordic hamstring exercise. Between-limb imbalance: calculated as left/right limb ratio, for the uninjured group and as uninjured/injured limb ratio, for the injured group. These between limbs imbalance ratios were them converted to the percentage difference using log transformed was data followed by back transformation. In some analysis, to have a single measure of eccentric knee flexor for each athlete, an averaging the peak forces from each limb (2-limb-average strength) was made (unilateral and bilateral analyses).
2017 van Dyk et al. [32]	Soccer (*n* = 413; M) Age: 26 ± 5 y Elite	Prospective cohort study	**8)** Eccentric knee flexor strength assessed with the device did not differ between injured and healthy players and do not provide a clinical value in predicting risk of HSI. Muscle strength is part from a multifactorial complex model that may lead to injury. **Device:** a custom-made uniaxial load cells (Delphi Force Measurement, Gold Coast, Australia; NordBord prototype). **Parameters:** peak force (highest); the best of 3 repetitions of Nordic hamstring exercise for each limb (left and right), reported in absolute terms (N) and relative to body weight (N/kg). Peak force (average); the mean of the peak forces from 3 contractions during Nordic hamstring exercise for each limb (left and right), reported in absolute terms (N) and relative to body weight (N/kg). Between-limb imbalance was measured as the left-right limb peak force ratio (bilateral and unilateral analyses).
2018 Chalker et al. [33]	Cricket (*n* = 44; M) Age: 18 ± 2 y U23 senior sub-elite and school level	Interventional cross-over study	**9)** The augmented feedback significantly increased mean eccentric knee flexor force, with the majority of this increase occurring in the weaker compared to stronger limb. A single session with the use of real-time visual feedback during the performance of the Nordic hamstring exercise did not improve between limb knee flexor strength asymmetries, but resulted in a non-significant, albeit small to moderate effect size decrease in between limb force asymmetries. **Device:** a custom-made uniaxial load cells (Delphi Force Measurement, Gold Coast, Australia; NordBord prototype). **Parameters:** peak force (average); the mean of the peak forces from 3 contractions during Nordic hamstring exercise for each limb (left and right), reported in absolute terms (N). Between-limb imbalance; calculated as a left/right limb ratio of the peak force (average), using log transformed raw data followed by back transformation. In some analysis, to have a single measure of eccentric knee flexor for each athlete, an averaging the peak forces from each limb (2-limb-average strength) was made (unilateral and bilateral analysis).
2018 Isik et al. [34]	Soccer (*n* = 88; M) Age: 16 ± 2 y U19-U14	Cross-sectional study	**10)** The athlete’s body weight affects strength imbalance, and young soccer players with reported lower extremity injuries in the previous 2 years had similar eccentric hamstring strength imbalance in comparison to non-injured soccer players. **Device:** NordBord Hamstring Testing device (Qutbluebox, Queensland, AUS). **Parameter:** between-limb imbalance; calculated as stronger leg peak force (N) − weaker leg peak force (N), with each leg force data being obtained from the best of 3 repetitions of Nordic hamstring exercise for each limb (left and right), reported in absolute terms (N) (unilateral analysis).
2018 van Dyk et al. [23]	Soccer (*n* = 288; M) Age: 25 ± 5 y Elite	Prospective cohort study	**11)** There was a substantial variability for isokinetic measures between seasons, as demonstrated by the large measurement error for all the contraction modes. A poor correlation was found between peak isokinetic hamstring eccentric torque and peak eccentric knee strength measured during Nordic hamstring exercise. Also, no correlation between bilateral imbalances measured in isokinetic strength testing and Nordic hamstring exercise testing was found. **Device:** a custom-made uniaxial load cells (Delphi Force Measurement, Gold Coast, Australia; NordBord prototype). **Parameter:** peak force (highest); the highest value obtained from 3 repetitions for each limb (left and right) in absolute terms (N), resulting in a left and right limb measure. Peak force (average); the mean of the peak forces from 3 contractions during Nordic hamstring exercise for each limb (left and right), reported in absolute terms (N). The peak force (highest) from Nordic hamstring exercise was correlated to the peak torque of isokinetic eccentric contraction at 60°/s (r = 0.35; r^2^ = 12%). The between-limb imbalance was calculated as a percentage following the formula left/right limb ratio of peak force.
2019 Franchi et al. [35]	Alpine Skiing (*n* = 170; 100 M, 70 F) Age: 14 ± 1 y Age: 22 ± 3 y Elite and U15	Cross-sectional study	**12)** There is a description of maximal eccentric hamstrings strength in alpine skiers from the youth to the elite level. This study highlights the importance of biological maturation regarding maximal eccentric hamstrings strength values in youth athletes, more specifically those who are close to their growth spurts. This study presents novel data that may offer novel insights for anterior cruciate ligament injury prevention in Alpine Skiing. **Device:** NordBord Hamstring Testing device (Vald Performance, Newstead, Australia). **Parameter:** all participants performed one set of 3 repetitions with 5–10 s of rest between repetitions. The peak force was considered the maximum value between the 3 repetitions, for the left and the right limb. The limbs’ asymmetry during testing was calculated as the difference between stronger and weaker leg expressed as a percentage. The parameters analyzed were both right and left limbs as well as limbs asymmetry.
2019 Hegyi et al. [27]	Soccer and Rugby (*n* = 13; M) Age: 23 ± 3 y Regional	Cross-sectional study	**13)** During Nordic hamstring exercise with hip flexed in 90°, there were higher eccentric knee flexor torque and lower hamstring EMG (biceps femoral long head and semitendinosus) levels compared to Nordic hamstring exercise with hip in neutral position in most phases of movement. There was similar peak eccentric knee flexor torque and EMG levels in Nordic hamstring exercise performed unilateral and bilateral modes, independently of hip position. During Nordic hamstring exercise with hip in neutral position, there was higher semitendinosus activity during the movement’s early phase and lower during movement’s final phase (toward full knee extension) than biceps femoral long head activity. During Nordic hamstring, exercise with hip flexed in 90°, there was higher semitendinosus activity than biceps femoral long head activity in the second half of bilateral movement and in the final phase of unilateral movement. **Device:** a device with load cells (ELAF, 1250 N; TE Connectivity, Schaffhausen, Switzerland) and a potentiometer (P4500; Novotechnik, Ostfildern, Germany). **Parameter:** peak torque (average); mean of instantaneous force in the dominant leg (N) multiplied by the lever arm (distance between femur’s lateral epicondyle and the center of the load cells) in meters, recorded in two repetitions.
2020 Giakoumis et al. [26]	Track and Field (*n* = 44; 23 M, 21 F) Age: 19–33 y Elite	Cross-sectional study	**14)** Male athletes produced greater absolute force levels compared to female athletes, but relative force (normalized to body weight) was similar between sexes. Long sprinters (400 m) presented stronger right leg than left leg, and short sprinters (100 m/200 m/110 m) showed similar strength between legs. There were no differences in eccentric hamstring strength between previously injured and uninjured athletes. **Device:** NordBord Hamstring Testing device (Qutbluebox, Queensland, AUS). **Parameter:** peak force (highest); the highest value obtained from 3 repetitions for each limb (left and right) in absolute terms (N) and relative to bodyweight (N/kg), resulting in a left and right limb measure. Peak force (average); the mean of the peak forces from 3 contractions during Nordic hamstring exercise for each limb (left and right), reported in absolute terms (N) and relative to bodyweight (N/kg). Peak torque (highest) (Nm); calculated by multiplying the length of the shank by the peak force (highest), reported in absolute terms (Nm) and relative to body weight (Nm/kg). The shank length (m) was determined as the distance from the lateral tibial condyle to the mid-point of the brace that was placed around the ankle during Nordic hamstring exercise. Between-limb imbalance: calculated by dividing torque or force of right limb by the measures of left limb. 0% indicates no imbalance, imbalance > 0% indicates more force/torque on the right side (unilateral and bilateral analysis)
2020 Markovic et al. [22]	Soccer (*n* = 155; M) Age: 11–17 y to 19–30 y Elite and U18	Cross-sectional study	**15)** The absolute and relative Nordic hamstring strength increases with players’ age, but this increase was not linear and there was an abrupt increase in Nordic hamstring strength in U16 category. In general, the body size is largely responsible for the observed age-related increase in absolute Nordic hamstring strength. Bilateral Nordic hamstring strength asymmetry varied non-significant (8–16%) between age groups, with the highest asymmetries being observed in U12 and U13 age groups (> 15%). There was a large negative correlation between eccentric knee flexor strength and sprint performance, with 27% spring performance variance being explained by relative Nordic hamstring strength (Nm/kg). **Device:** a custom-made uniaxial load cells (FL34-100 kg; Forsentek Co., Shenzhen, China; NordBord prototype). **Parameter:** peak force (highest): the highest value obtained from 3 repetitions for each limb (left and right) in absolute terms (N) and relative to bodyweight (N/kg), resulting in a left and right limb measure. Peak torque (highest): the product of peak force by the shank length (m), left and right, in absolute terms (Nm) and relative to body weight (Nm/kg). The shank length (m) was determined as the distance from the lateral tibial condyle to the mid-point of the brace that was placed around the ankle during Nordic hamstring exercise. The bilateral strength asymmetry of knee eccentric flexor strength was expressed in % following the formula: 1 − (dominant leg strength/non-dominant leg strength) × 100. In some analysis, to have a single measure of eccentric knee flexor for each athlete, an averaging the peak forces or peak torque from each limb (2-limb-average strength) was made (unilateral and bilateral analysis).
2020 Ribeiro-Alvares et al. [25]	Soccer (*n* = 210; M) Age: 24 ± 5 y Elite	Cross-sectional study	**16)** Previously injured players presented strength deficit on injured limb in relation to their contralateral limb and to the uninjured player’s limb. Previously injured and uninjured players displayed similar between-limb asymmetry values, but half of the previously injured players and 37% of uninjured players presented between limb strength asymmetry > 10%. **Device:** device was based on the prototype validated by Opar et al. (2013) with two independent commercially available load cells (Elastic, E-sporte Soluções Esportivas, Brasilia, Brazil). **Parameter:** peak force (highest); the highest value obtained from 3 repetitions for each limb (left and right) in absolute terms (N), resulting in a left and right limb measure. Between-limb imbalance: calculated as the player’s stronger limb minus the weaker limb with the stronger limb being used as the reference value (i.e., 100%). For the injured group, limbs were analyzed separately; for the control group, the eccentric knee flexor peak force was average between limbs (bilateral and unilateral analyses).
2020 Vicens-Bordas et al. [24]	Soccer (*n* = 284; M) Age: 23 ± 4y Regional	Cross-sectional study	**17)** Athlete’s age was negative associated with preseason eccentric hamstring strength with a mean reduction on knee flexor strength of 0.9% per year increased. Players with previous hamstring injury duration of more than 3 weeks had 9% lower preseason knee flexor eccentric strength compared to players with no previous hamstring injury. **Device:** a custom-made uniaxial load cells (Delphi Force Measurement, Gold Coast, Australia; NordBord prototype). **Parameter:** peak force (average); the mean of the peak forces from 3 contractions during Nordic hamstring exercise for each limb (left and right), reported in relative terms (N/kg). Between-limb imbalance: analyzed using the formula: (strongest limb − weakest limb)/(sum of both limbs) (bilateral and unilateral analyses).

Cross-sectional: consist in assessing a population at a single point in time, cannot demonstrate temporality, therefore can present prevalence and associated factors [36]

Interventional cross-over study: interventional study where participants underwent for all treatment’s arms

Prospective cohort study: it is suggested to be the gold standard of observational research and can identify the potential risk factors for an injury or disease, due to the temporality [36]

Reliability and case-control study: in a reliability study design, researchers are interested in measuring the consistency of some measure across time, the consistency of people’s responses on a measure, and the consistency of different observers and their judgments of some measures. Case-control study designs refers to retrospective observational study in which participants are identified and selected based on their case status (i.e., injured or not injured); this type of study allows the establishment of a statistical association between the exposure for some variables and outcomes [36]

*n* number of subjects, *F* female, *M* male, *y* years old, *N* Newton, *BM* body mass, *Kg* kilogram, *HSI* hamstring strain injury, *ICC* intraclass correlation coefficient, *SEM* standard error of measurement

The selected studies were related to hamstring (*n* = 10) and anterior cruciate ligament injuries risks (*n* = 1), previous experience effect (*n* = 1), device reliability (*n* = 1), body mass effect (*n* = 2), augmented feedback (*n* = 1), sprint performance (*n* = 1), variability and correlation with isokinetic strength testing (*n* = 1), and body positioning during the test (*n* = 1). In addition, 18 parameters related to eccentric hamstring strength were obtained from the devices using 3-to-6 unilateral or bilateral trials: peak force (average or highest, and absolute or relative), peak torque (average or highest, and absolute or relative), average force (N), average power (W), peak power (W), average velocity (m/s), peak velocity (m/s), and between-limb imbalance. The between-limb imbalance included 5 different equations: (1) left-right limb ratio, 2) stronger leg peak force − weaker leg peak force (× 100); (3) 1 − (dominant leg strength/non-dominant leg strength) × 100; (4) stronger limb minus the weaker limb, with the stronger limb being used as the reference value, i.e., 100%; and (5) (strongest limb − weakest limb)/(sum of both limbs)]. However, the same parameters were calculated in different ways in different studies (see details in Table 2).

## Discussion

The purpose of this systematic review was to analyze the findings related to the evaluation of hamstrings eccentric strength with alternative devices. Thus, we described the instruments used to assess eccentric hamstring strength and the clinical outcomes related. There are four different devices used to evaluate eccentric knee flexors strength data. Eccentric hamstring strength using these devices was evaluated from 18 different parameters obtained in 3–6 trials. There is inconsistent evidence correlating eccentric knee flexor strength with an increased risk of sustaining hamstring strain injuries, as well as limited evidence associating eccentric knee flexors strength and sports performance.

The first study found in the literature reported that athletes using an instrumented flywheel leg-curl machine showed greater eccentric strength performances [14]. However, this finding may also suggest an influence of the learning effect [37, 38]. Therefore, a previous execution of the test procedures to reduce the intra-subject variability, which is called familiarization, is therefore necessary and constitutes an important factor to be considered in this context [39, 40]. Augmented feedback, with its effects well recognized in the scientific literature [41, 42], was tested in another study using a single session of real-time visual feedback during the Nordic hamstring testing. With this procedure, the mean peak force was increased [33]. Thus, researchers and practitioners should pay attention to the importance of familiarization sessions and whether augmented feedback is being used by athletes, to guarantee higher levels of reliability of the measures obtained with these devices.

The NordBord device [15, 16, 22–24, 26, 28–35] and another similar device [25] were the most common instruments used for assessing eccentric hamstring strength. These devices have been developed as low-cost alternatives to the widespread used isokinetic dynamometers [12] and the technically limited handheld dynamometers [13]. The NordBord device for bilateral assessment of Nordic hamstring exercise showed a high to moderate test-retest reliability, i.e., intraclass correlation coefficient (ICC) = 0.83–0.90 and standard error of measurement (SEM) = 6–9%. However, a lower reliability was found during unilateral testing (ICC = 0.56–0.73 and SEM = 10–11%) [15]. Also, for between-limb imbalance, it was found that only the ratios based on the peak force averaged across 6 trials had acceptable reliability values (ICC = 0.85; 95% CI 0.71–0.93; SEM = 5%, 95% CI 4–6%) [15]. Despite the good reliability of some NordBord measures, a poor correlation was found between peak isokinetic hamstring eccentric torque (60°/s) and forces measured with the NordBord device (*r* = 0.35; *r*^2^ = 12%) [23]. Besides, there was no correlation between bilateral imbalances in isokinetic eccentric contraction strength (60°/s) and eccentric strength measured during the Nordic hamstring exercise [23], despite the former being considered the gold standard [43] and its use to determine quadriceps-to-hamstring ratios aiming to identify risk profiles [44]. The authors also reported a substantial variability for isokinetic measures between different seasons with the SEM ranging 15–19% [23]. Additionally, during the Nordic hamstring exercise with the hip in neutral position, there was a higher semitendinosus activity in the early phase of the movement and a higher biceps femoral long head activity during the final phase. This behavior was changed with hip flexed at 90°, where a higher semitendinosus activity was found in the final phase of the movement [27]. During the Nordic hamstring exercise, the athlete is required to resist via forceful eccentric knee flexor contractions, thus increasing the external torque around the knee joint as the athlete progresses toward the ground. In contrast, isokinetic testing imposes maximal effort throughout the full range of motion [28]. The poor correlation of imbalance measures between both tests may be justified by the fact that the Nordic hamstring exercise only measures the imbalance when both legs are tested at the same time [45]. In contrast, during isokinetic testing, each leg is evaluated separately. Therefore, bilateral testing should be preferred when evaluating the Nordic hamstring exercise with these devices.

It has been previously suggested that athletes with a history of hamstring injuries present knee flexor weakness in their injured limb when compared to their uninjured limb and to uninjured athletes [15, 25]. In the same manner, players with hamstring injury lasting more than 3 weeks, exhibited a ~ 9% lower knee flexor eccentric strength than uninjured players during the preseason [24]. Contrary to this, it has been also observed no difference in eccentric hamstring strength between previously injured and uninjured athletes [26]. Furthermore, previously hamstring injured and uninjured athletes have displayed similar between-limb asymmetries on eccentric knee flexors strength, with half of the previously injured athletes and 37% of the uninjured athletes presenting between-limb asymmetries above ~ 10% [25]. In this regard, players with previous lower extremity injuries had shown similar eccentric strength imbalances in comparison to non-injured players, with athletes’ body masses being positively correlated to bilateral eccentric strength imbalances [34]. This conflicting evidence was also found in prospective cohort studies. Previously, some authors have suggested that levels of eccentric knee flexors strength below 256 N at the start of pre-season, 279 N at the end of pre-season in Australian Rules footballers [28], and 337 N for the whole season in soccer players [31] may increase the risk of a hamstring strain injury by ~ 4.4 times [31]. Furthermore, higher levels of between-limb imbalance (> 15%) were also associated with an increased risk of hamstring strain injury [29]. Conversely, others did not find any association between eccentric hamstring strength or between-limb imbalances and an increased risk of hamstring strain injuries [28, 29, 31, 32]. However, it is noteworthy that these previous studies evaluated players from different sports. Therefore, it is possible that the differences between physical characteristics of these athletes [46, 47] and the specific demands of each sport could explain the inconsistencies observed in these previous studies [29, 48, 49]. Moreover, these previous studies looked for this association in a linear fashion while isolating specific parameters. Since hamstring injuries have a multifactorial nature, it may be suggested for future studies to better test this association through the combination of different risk factors, including sports performance, running demands, and hamstrings strength [50].

Another reason for the inconsistencies observed in previous literature could be the different characteristics between hamstring demands during running activities and the Nordic hamstring exercise. Although the exact mechanisms for hamstring injuries are not fully understood [50, 51], it is suggested to occur mainly during the late swing phase or the early stance phase, in which hamstrings are highly activated at longer lengths [51–55]. Hamstrings are required to contract eccentrically during the terminal swing phase of a running gait cycle to decelerate the forward swinging shank and to oppose the external hip-flexor and knee extensor torques developed [56–58]. In addition, they are required to contract concentrically during early stance to absorb the high ground reaction forces [59]. Further, hamstrings demands increase with higher running speeds, such as sprinting at maximal or close to maximal speeds [54, 59, 60], which is the situation at which the hamstrings strain injuries frequently occur [53, 61]. However, eccentric hamstring strength assessment with these devices occurs at the slowest possible knee angular velocity [15, 31], with trials performed at very low angular velocities of − 30°s^−1^ [62]. Furthermore, it has been also shown that when running at ~ 75% of the maximal running speed, there was approximately a 15% higher hamstrings electromyographic (EMG) activity than during a maximum voluntary isometric contraction [63] and higher EMG amplitudes when compared to the Nordic hamstring exercise [63, 64]. Therefore, the strength measures assessed during the Nordic hamstring exercise do not elicit the same demands as during running activities. In addition, some evidence have pointed out that low levels of eccentric strength may reduce the hamstring ability to perform well during the gait cycle, potentially increasing the hamstring strain injury risk [52, 65]. While we are not questioning the well-established effects of the Nordic hamstring exercise on reducing hamstring strain injury risk [66–69], it is important to consider that hamstring strain injuries are multifactorial and complex [51, 70–72], and therefore, caution should be taken by practitioners when using some isolated measurements, such as peak forces or between-limb imbalances during this test in an attempt to estimate a hamstring strain injury risk.

The horizontal component of the ground reaction forces is suggested to be the key mechanical feature of sprint acceleration performance [73–77], with subjects who are able to produce the greatest amounts of horizontal force in sprint running presenting higher eccentric hamstring peak torque capability and also being able to activate to a greater extent their hamstring muscles before initial ground contact [75]. Of note, this parameter can be monitored with other new technologies not included in the current review (i.e., mobile app and sports radar system) [77, 78]. In addition, the contributions of horizontal and vertical components of ground reaction forces on sprint performance have been described in the literature to be 17– 61% and 17–23%, respectively [73, 76, 79, 80]. In this regard, limited evidence indicated that eccentric hamstring strength measured with the NordBord device was largely correlated to 20-m sprint performance (*r* = − 0.52; *r*^2^ = 27%, *p* < 0.05) [22]. Therefore, all this information suggests that higher levels of eccentric hamstring strength could be an important component of an athlete’s neuromuscular capabilities to achieve higher speeds and thus a better sprint performance [22, 75]. Notably, the 20-m sprint time and the eccentric hamstring strength measured in the Nordic hamstring exercise share a common variance of 27% in youth soccer players [22]. Thus, sports practitioners interested in monitoring the neuromuscular status during the training process could use these tools to evaluate the eccentric strength of hamstring muscles in the same manner as vertical jump evaluations. This suggestion is based on the association levels observed between sprint performances and vertical jump height in elite sprinters (*r*^2^ = 47 to 76%) [81], collegiate soccer players (*r*^2^ = 22 to 46%) [82], and elite young basketball players (*r*^2^ = 37%) [83]. Therefore, these devices may serve as an appealing option for neuromuscular status monitoring, as occurs with vertical jump performance evaluations [84].

Attention should be given to how the eccentric hamstring strength parameters evaluated by these devices were calculated in the articles included. In an instrumented flywheel leg-curl device, the parameters were calculated from 6 coupled concentric-eccentric actions [14]. During Nordic hamstring exercises, some studies adopted the peak force averaged from 3 [23, 24, 26, 28–33] or 6 [15] bilateral maximal repetitions in absolute values (N) [15, 23, 26, 28–33], while others reported these values normalized by body mass (N/kg) [24, 26, 28, 31, 32]. There were also reports on these values relative to moment of the season, expressed as the late preseason/early preseason ratio [30]. Some researchers used the highest peak force of 3 repetitions in absolute values (N) [16, 22, 23, 25, 26, 29, 32, 35] or normalized by body mass [22, 26, 29, 32], while others adopted the highest peak force of 6 repetitions in absolute terms (N) [15]. Some authors measured eccentric hamstring torque through the product of mean peak force data (N) [27, 31] or the highest peak force data (N) [22, 26] by the lever arm of shank length, with this measure reported in absolute terms (Nm) [22, 26, 27, 31] or normalized by body mass (Nm/kg) [22, 26, 31]. All these inconsistencies were also presented for between-limb imbalance measures, calculated as percentage differences of left/right limb force ratio for uninjured players and uninjured/injured limbs force ratio in the injured group [28, 29, 31]. In other studies, it was calculated as the left-right limb force ratio [32], converted to the percentage difference using log-transformed raw data followed by back transformation [15, 33], as the stronger leg peak force minus weaker leg [25, 34, 35], as the stronger limb being used as the reference value (i.e., 100%) [25], or by dividing torque or force of the right limb by the measures of left limb [26]. There was also one study that calculated Eq. 1 [24]:

1 $$ \left(\mathrm{strongest}\ \mathrm{limb}-\mathrm{weakest}\ \mathrm{limb}\right)/\mathrm{sum}\ \mathrm{of}\ \mathrm{both}\ \mathrm{limb}\mathrm{s} $$

and another that adopted Eq. 2 [22]:

2 $$ \left(\mathrm{dominant}\ \mathrm{leg}\ \mathrm{strength}/\mathrm{non}-\mathrm{dominant}\ \mathrm{leg}\ \mathrm{strength}\right)\ast 100 $$

All this variety makes it difficult to compare the different results observed in studies examining lower limbs imbalance with regard to hamstring strain injury or sports performance.

Lastly, although with a limited number of athletes for sex comparisons, male athletes were found to produce greater absolute force levels compared to female athletes, but the relative force (normalized to body mass) was similar between sexes [26]. This is important as eccentric hamstring strength assessed by these devices is suggested to be body mass dependent [16, 22]. Additionally, Nordic hamstring strength increases with the player’s age, and body size has been reported to be largely related to the increase in absolute Nordic hamstring strength [22]. Therefore, adopting absolute units (i.e., N and Nm) or simply dividing eccentric hamstring strength by body mass (i.e., N/Kg; Nm/kg) may not be optimal strategies. In this regard, future studies may use allometric scaling for solving this issue. An alternative method could be to apply a correction factor, as previously suggested [16] with the following Eq. (3):

3 $$ \mathrm{eccentric}\ \mathrm{strength}\ \left(\mathrm{N}\right)=4\ast \mathrm{body}\ \mathrm{mass}\ \left(\mathrm{kg}\right)+26.1 $$

Finally, it is worth noting that in the first studies found in the scientific literature, 6 repetitions were performed for the hamstring assessments [14, 15]. Among these studies, in a single reliability study, the authors reported that, when peak force was averaged across the 6 trials, the results were more reliable [15]. This finding agrees with previous studies that verified greater sensitivity for the averaged results than for the highest performance in trials for detecting trained related changes in countermovement jumps [84, 85]. Future studies should consider a standard method to assess eccentric knee flexors strength during the Nordic hamstring exercise, including 6 trials and body mass normalization. On the other hand, from a clinical perspective, these measures should be used over the season thus allowing the management of training loads and also as criteria before returning to play. This information could guide sports professionals during the training and rehabilitation processes to decrease injury and re-injury risks.

## Conclusions

There is a growing scientific evidence suggesting the use of new devices to evaluate the eccentric hamstring strength of athletes. These devices have been developed as low-cost alternatives to the widespread used isokinetic dynamometers and the technically limited handheld dynamometers. There is inconsistent evidence correlating eccentric knee flexor strength with an increased injury risk of hamstrings, and very limited evidence about its relationship with athletic performance. The parameters most widely used during these evaluations were peak force (average or highest), peak torque (average or highest), and between-limb imbalance (left-right limb ratio). Due to the variety of strength measurements observed in the current literature, there is an urgent need for better defining the procedures and the parameters to be used with these devices. The eccentric strength of hamstrings assessed by these devices may be useful as a tool for monitoring the neuromuscular status of athletes during a whole season.

## Supplementary Information

**Additional file 1.**

## Data Availability

After publication, all data necessary to understand and assess the conclusions of the manuscript are available to any reader of Sports Medicine-Open.

## Abbreviations

BM: Body mass
EMG: Electromyography
F: Female
HSI: Hamstring strain injury
ICC: Intraclass correlation coefficient
M: Male
*n*: Sample size
PRISMA: Preferred Reporting Items for Systematic Reviews and Meta-Analyses
*r*^2^: Coefficient of determination
SEM: Standard error of measurement
y: Years old
