# Supplementary material for: Eccentric Strength Assessment of Hamstring Muscles with New Technologies: a Systematic Review of Current Methods and Clinical Implications
Source: Sports Med Open. 2021 Jan 28;7:10. doi: 10.1186/s40798-021-00298-7 (PMC7843797; doi:10.1186/s40798-021-00298-7)
Supplement: Supplementary file 1 — Additional file 1. [file 40798_2021_298_MOESM1_ESM.pdf]

	A	B	C	D	E	∑
2006 Tous-Fajardo et al. [14]	1	1	1	1	1	5
2013 Opar et al. [15]	1	2	1	2	1	7
2015 Bourne et al. [29]	1	2	2	2	1	8
2015 Opar et al. [28]	1	2	2	2	1	8
2015 Opar et al. [29]	1	2	2	2	1	8
2016 Buchheit et al. [16]	1	2	2	2	1	8
2016 Timmins et al. [31]	1	2	2	2	1	8
2017 van Dyk et al. [32]	1	2	2	1	1	7
2018 Chalker et al. [33]	1	2	2	2	1	8
2018 Isik et al. [34]	1	2	2	2	1	8
2018 van Dyk et al. [23]	1	2	2	1	1	7
2019 Francis et al. [35]	1	2	2	2	1	8
2019 Hegyi et al. [27]	1	1	2	2	1	7
2020 Giakoumis et al. [26]	1	2	2	2	1	8
2020 Markovic et al. [22]	1	2	2	2	1	8
2020 Ribeiro-Alvares et al. [25]	1	2	2	2	1	8
2020 Vicens-Bordas et al. [24]	1	2	2	2	1	8
					Average	7.6

	Criteria	Definition	Scoring 0	1	2
A	Peer reviewed	Study published in peer-reviewed journal	No	Yes	-
B	Number of participants	Number of participants included in study findings	<5	6-30	>31
C	Population defined	Age, sex, sport, experience time (or level) were described	No	Partly	Yes
D	Experimental design	Experimental design the study period was described and replicable	No	Partly	Yes
E	Eccentric strength parameters	The eccentric strength parameters of hamstring assessed by devices were described	No	Yes	-

Eccentric strength assessment of hamstring muscles with new technologies: a systematic review of current methods and clinical implications

João Gustavo Claudino; Carlos Alberto Cardoso Filho; Natália Franco Netto Bittencourt; Luiz Guilherme; Crislaine Rangel Couto; Roberto Chiari Quintão; Guilherme Fialho; Otaviano de Oliveira Júnior; Alberto Carlos Amadio; Daniel Boullosa; Júlio Cerca Serrão. Sports Medicine. Corresponding author: João Gustavo Claudino; claudinojgo@usp.br. Universidade de São Paulo, School of Physical Education and Sport - Laboratory of Biomechanics.
